# HCMMD: systematic evaluation of metabolites in body fluids as liquid biopsy biomarker for human cancers

**DOI:** 10.18632/aging.205779

**Published:** 2024-04-26

**Authors:** Xun Dong, Yaoyao Qu, Tongtong Sheng, Yuanming Fan, Silu Chen, Qinbo Yuan, Gaoxiang Ma, Yuqiu Ge

**Affiliations:** 1 State Key Laboratory of Natural Medicines, School of Traditional Chinese Pharmacy, China Pharmaceutical University, Nanjing, China; 2 School of Public Health, Nanjing Medical University, Nanjing, China; 3 Department of Urology, Wuxi Fifth People’s Hospital, Wuxi, China; 4 The Clinical Metabolomics Center, China Pharmaceutical University, Nanjing, China; 5 Deparment of Oncology, Pukou Hospital of Chinese Medicine affiliated to China Pharmaceutical University, Nanjing, China; 6 Department of Public Health and Preventive Medicine, Wuxi School of Medicine, Jiangnan University, Wuxi, China

**Keywords:** cancer, liquid biopsy, metabolites, diagnostic biomarker, database

## Abstract

Metabolomics is a rapidly expanding field in systems biology used to measure alterations of metabolites and identify metabolic biomarkers in response to disease processes. The discovery of metabolic biomarkers can improve early diagnosis, prognostic prediction, and therapeutic intervention for cancers. However, there are currently no databases that provide a comprehensive evaluation of the relationship between metabolites and cancer processes. In this review, we summarize reported metabolites in body fluids across pan-cancers and characterize their clinical applications in liquid biopsy. We conducted a search for metabolic biomarkers using the keywords (“metabolomics” OR “metabolite”) AND “cancer” in PubMed. Of the 22,254 articles retrieved, 792 were deemed potentially relevant for further review. Ultimately, we included data from 573,300 samples and 17,083 metabolic biomarkers. We collected information on cancer types, sample size, the human metabolome database (HMDB) ID, metabolic pathway, area under the curve (AUC), sensitivity and specificity of metabolites, sample source, detection method, and clinical features were collected. Finally, we developed a user-friendly online database, the Human Cancer Metabolic Markers Database (HCMMD), which allows users to query, browse, and download metabolite information. In conclusion, HCMMD provides an important resource to assist researchers in reviewing metabolic biomarkers for diagnosis and progression of cancers.

## INTRODUCTION

Cancer is a major global public health problem, with a significant impact on mortality worldwide. In 2020, an estimated 10 million people died of cancer globally [1]. Early diagnosis is crucial for effective cancer management and better prognosis. However, approximately 50% of cancers are diagnosed at advanced stages [2, 3]. Effective treatment of advanced cancer often involves the use of modern systemic and targeted drugs, which can be costly and may have limited efficacy [4]. Early cancer detection has been shown to provide substantial health benefits, including increased survival rates and reduced morbidity [2]. Although several blood-based biomarkers, such as carcinoembryonic antigen (CEA) and prostate-specific antigen (PSA), have been used for cancer screening in the past few decades, their sensitivity and specificity have been found to be unsatisfactory, limiting their effectiveness [5]. Therefore, there is a pressing need to identify biomarkers that exhibit high sensitivity and specificity for the early detection of cancer.

Metabolomics, which involves the comprehensive analysis of small molecule metabolites in cells, tissues, or whole organisms, has undergone rapid technological evolution in the past two decades [6–8]. By measuring downstream chemical phenotypes of genomic, transcriptomic, and proteomic variability, metabolomics can provide a more comprehensive understanding of the biological system [6, 9, 10]. Research has shown that metabolites play a crucial role in various diseases such as obesity, diabetes, cardiovascular disease, respiratory conditions, and cancer [6, 11]. Metabolomics has emerged as an accurate and non-invasive diagnostic tool, accompanied by the development of novel and sensitive measurement techniques [12, 13]. The uncontrolled proliferation of tumor cells requires metabolic regulation [14–16], and metabolic reprogramming is a hallmark of malignancy [17]. In recent years, several highly sensitive and specific metabolic biomarkers have been identified in liquid biopsy studies. For instance, Sreekumar et al. reported that sarcosine had a diagnostic value with an AUC of 0.69 (95% CI: 0.55, 0.84) for prostate cancer [18]. Soga et al. discovered that serum γ-glutamyl dipeptides had an AUC of 0.76 for hepatocellular carcinoma [19]. Tyrosine and glutamine-leucine in serum had an AUC of 0.98 for the diagnosis of colorectal cancer [20]. N^1^, N^12^-diacetylspermine in serum had an AUC of 0.65 (95% CI, 0.59 to 0.72) for the diagnosis of non–small-cell lung cancer [21]. The AUC value of creatine nucleoside in urine was 0.79 for differential diagnosis between adrenocortical carcinoma and benign adrenal tumors [22].

This study aimed to comprehensively evaluate the role of metabolites in cancers. Cancer-related metabolites were searched from the PubMed database. The collected information includes cancer types, sample size, HMDB ID, metabolic pathway, area under the curve (AUC), sensitivity, specificity, sample source, detection method, and clinical features. Importantly, a user-friendly online database was developed, named Human Cancer Metabolic Markers Database (HCMMD), to assist users in querying, browsing, and downloading information about the cancer-related metabolites.

## Advances in metabolomics

Metabolomic analysis is a technique used to analyze the type and content of small molecule metabolites in biological samples [23]. Four major technologies are commonly used for metabolomics: gas chromatography mass spectrometry (GC-MS) [24], liquid chromatography mass spectrometry (LC-MS) [25], capillary electrophoresis mass spectrometry (CE-MS) [26], and nuclear magnetic resonance spectroscopy (NMR) [27]. These techniques can assess changes in metabolic processes and provide a summary of alterations at the DNA, RNA, and protein levels [28]. Metabolomics has been used to reveal the mechanisms of basal metabolic processes in diseases [29, 30], and in some cases, it may be the most sensitive method for identifying the pathological state of cancer patients, as even small changes in gene or protein expression can lead to remarkable changes in protein activity and metabolite levels [31–33]. Metabolomics can provide an effective method for screening for cancer, guiding treatment strategies, assessing efficacy, and tracking cancer progression [34–37]. Additionally, it can help to identify therapeutic targets and promote drug discovery [38, 39].

## Metabolism-related biomarkers for cancers

### Database establishment

**Figure 1:**
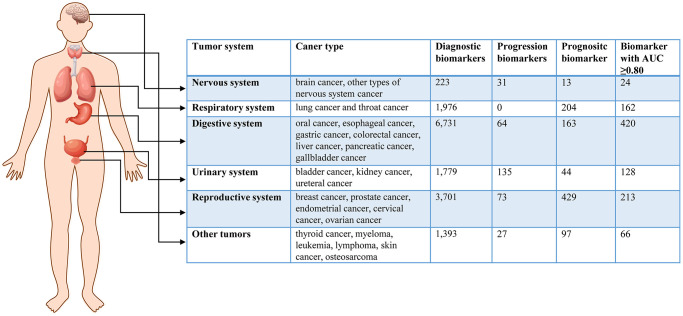
The basic information of metabolic biomarkers in different system cancers.

**Figure 2:**
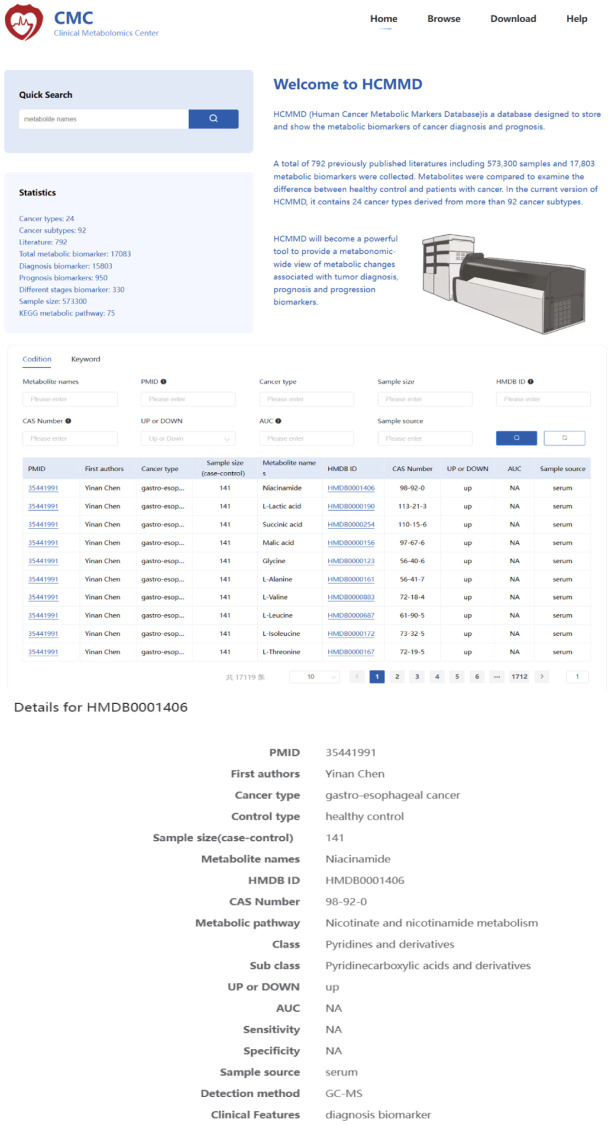
The web interface of the HCMMD database.

To search for cancer-related metabolites from PubMed, we used the keywords “metabolomics” OR “metabolite” AND “cancer”. The eligible data included 22,254 articles published before July 30, 2022. We selected literature that focused on human tumors, samples from liquid biopsies, and individual biomarkers with diagnostic, prognostic, and predictive value. After filtering the results, we identified 792 studies with a total of 573,300 samples and 17,083 metabolic biomarkers. The included studies covered 24 types of cancer derived from 92 subtypes. We recorded information such as cancer type, sample size, HMDB ID, metabolic pathway, AUC, sensitivity, specificity, sample source, detection method, and clinical features. Figure 1 summarizes the basic information and diagnostic value of metabolic biomarkers in different cancers. Importantly, we created a new online database, called the HCMMD, which allows users to explore and analyze cancer-related metabolic biomarkers (Figure 2).

### Digestive system tumors

Digestive system cancer encompasses oral cancer, esophageal cancer, gastric cancer, colorectal cancer, liver cancer, pancreatic cancer, and gallbladder cancer. The five-year survival rates for liver cancer and pancreatic cancer are only 20% and 11%, respectively [40]. A total of 315 articles were collected, covering 7 cancer types and 23 subtypes, with sample sizes ranging from 9 to 3,109 [20, 41]. The reports included 6,731 diagnostic biomarkers, 64 progressive biomarkers, and 163 prognostic biomarkers. Of these biomarkers, 3,609 were from serum samples, 1,619 from plasma samples, 974 from urine samples, and 460 from saliva samples. Among the biomarkers, 1,763 belonged to amino acids and 246 belonged to bile acids. Several studies have shown that bile acids are closely related to digestive system tumors [23, 42, 43]. Of the biomarkers, 79 had an AUC ≥0.95, and 420 had an AUC ≥0.80.

**Figure 3:**
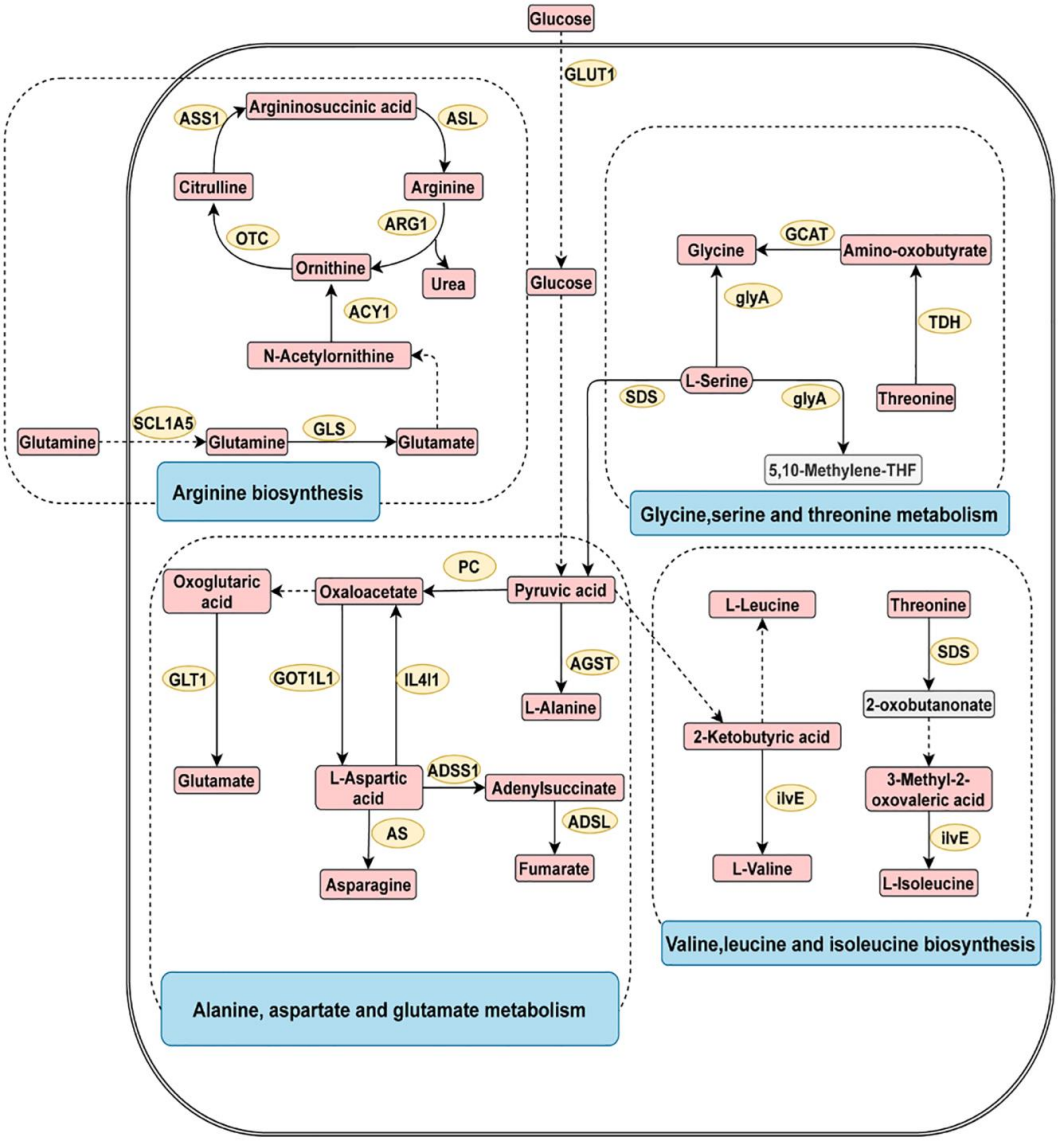
**The four most related metabolic pathways in digestive system tumors.** The metabolic process of metabolites and the proteins involved in their regulation. Differentially expressed metabolites (red), unchanged metabolites (grey), regulated proteins (yellow), metabolic pathway names (blue). Abbreviations: ACY1: aminoacylase; ADSL: adenylosuccinate lyase; ADSS1: adenylosuccinate synthase 1; AGST: alanine-glyoxylate transaminase; ARG1: arginase 1; AS: asparagine synthase; ASL: argininosuccinate lyase; ASS1: argininosuccinate synthase 1; GCAT: glycine C-acetyltransferase; GLS: glutaminase; GLT1: glutamate synthase; GLUT1: glucose transporter 1; glyA: glycine hydroxymethyltransferase; GOT1L1: glutamic-oxaloacetic transaminase 1 like 1; ilvE: branched-chain amino acid aminotransferase; IL4I1: interleukin 4 induced 1; OTC: ornithine transcarbamylase; PC: pyruvate carboxylase; SDS: threonine ammonia-lyase; SCL1A5: solute carrier family 1; TDH: threonine 3-dehydrogenase.

To explore the underlying pathogenesis of digestive system cancer, metabolite pathway enrichment analysis was conducted (Supplementary Figure 1). As shown in Figure 3, the four most closely related pathways for digestive system cancer were glycine serine and threonine metabolism, arginine biosynthesis, alanine aspartate and glutamate metabolism, and valine leucine and isoleucine biosynthesis.

### Reproductive system tumors

Reproductive system cancer comprises breast cancer, prostate cancer, endometrial cancer, cervical cancer, and ovarian cancer. Among all cancers, new cases of reproductive system cancer rank first [40]. In this review, we collected 238 articles covering 5 cancer types and 14 subtypes. The minimum sample size was 12 [44], and the maximum sample size was 6,114 [45]. These studies included 3,701 diagnostic biomarkers, 73 progression biomarkers, and 429 prognostic biomarkers. Of these metabolic markers, 1,462 biomarkers were from serum samples, 1,715 were from plasma samples, and 750 were from urine samples. Among the metabolic markers, 1,044 belonged to amino acids. A total of 213 diagnostic markers had an AUC ≥0.80.

**Figure 4:**
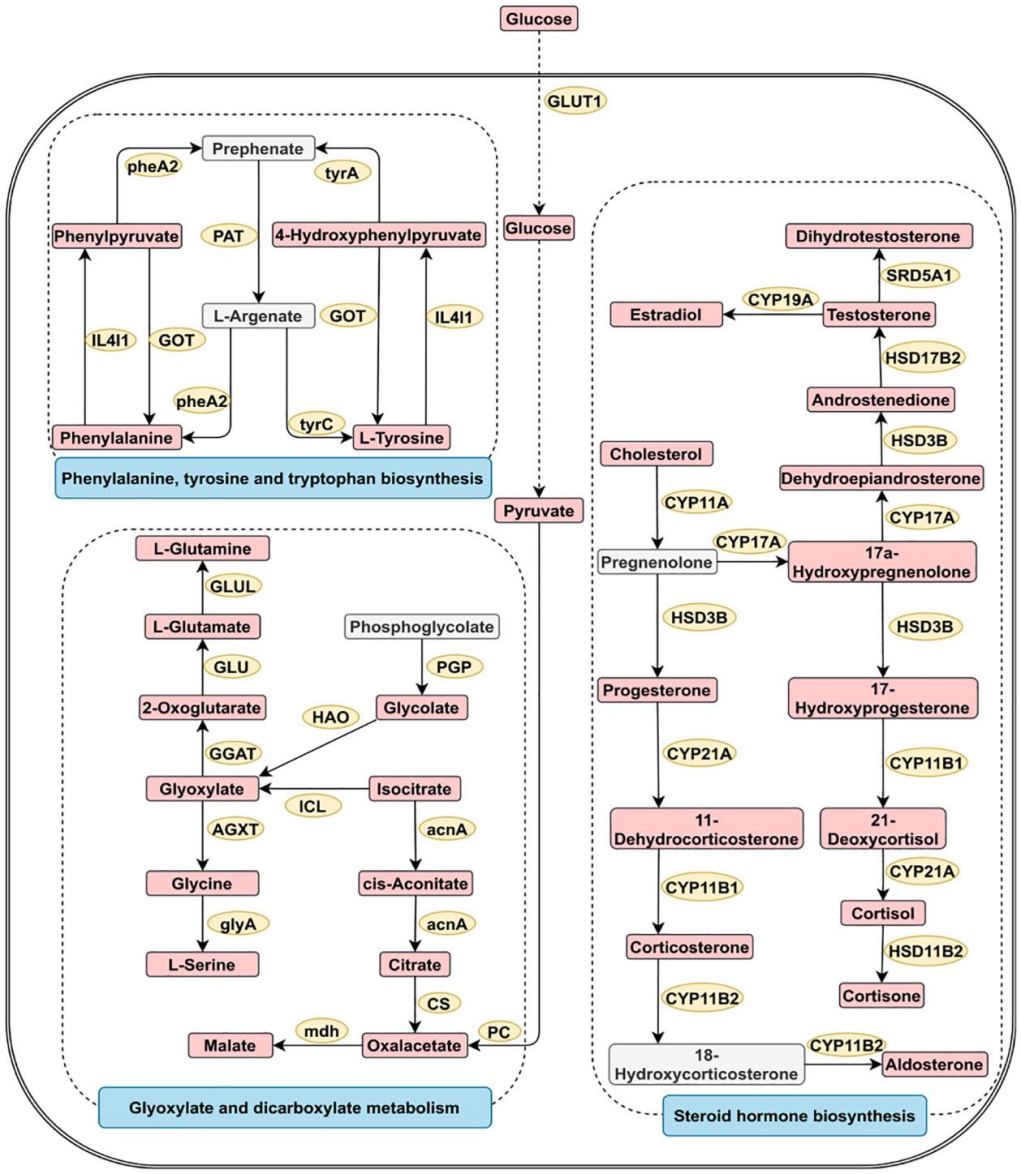
**Three greatly important metabolic pathways in digestive system tumors.** The metabolic process of metabolites and the proteins involved in their regulation. Differentially expressed metabolites (red), unchanged metabolites (grey) regulated proteins (yellow), metabolic pathway names (blue). Abbreviations: acnA: aconitate hydratase; AGXT: alanine-glyoxylate transaminase/serine-glyoxylate transaminase and serine-pyruvate transaminase; CS: citrate synthase; CYP11A: cholesterol monooxygenase; CYP17A: steroid 17alpha-monooxygenase/17alpha-hydroxyprogesterone deacetylase; CYP19A: aromatase; CYP11B1: steroid 11beta-monooxygenase; CYP11B2: steroid 11beta-monooxygenase/corticosterone 18-monooxygenase; CYP21A: steroid 21-monooxygenase; GGAT: glutamate--glyoxylate aminotransferase; GLU: glutamate synthase; GLUL: glutamine synthetase; GLUT1: glucose transporter 1; glyA: glycine hydroxymethyltransferase; GOT: aspartate aminotransferase; HAO: (S)-2-hydroxy-acid oxidase; HSD17B2: corticosteroid 11-beta-dehydrogenase isozyme 2; HSD17B2: 17beta-estradiol 17-dehydrogenase/3alpha(17beta)-hydroxysteroid dehydrogenase; HSD3B: 3beta-hydroxy-Delta5-steroid dehydrogenase; IL4I1: L-amino-acid oxidase; ICL: isocitrate lyase; mdh: malate dehydrogenase; PAT: bifunctional aspartate aminotransferase and glutamate/aspartate-prephenate aminotransferase; PC: pyruvate carboxylase; PGP: phosphoglycolate phosphatase; pheA2: prephenate dehydratase; SRD5A1: 3-oxo-5-alpha-steroid 4-dehydrogenase 1; tyrA2: prephenate dehydrogenase; tyrC: cyclohexadieny/prephenate dehydrogenase.

Metabolite pathway enrichment analysis of diagnostic metabolites was performed for reproductive system cancers (Supplementary Figure 2). The four most closely related pathways for reproductive system cancer were glycine serine and threonine metabolism, alanine aspartate and glutamate metabolism, arginine and proline metabolism, and valine leucine and isoleucine biosynthesis. Figure 4 shows the three most significant pathways involved in reproductive system cancer.

### Respiratory system tumors

Respiratory system cancer includes lung cancer and throat cancer. Lung cancer is the leading cause of cancer-related morbidity and mortality worldwide, with a five-year relative survival rate of only 22% [1, 40]. In this review, we collected 102 publications on respiratory system tumors, including 2 cancer types and 8 cancer subtypes. The sample size ranged from 14 to 1,196 [46, 47]. These publications included 1,976 diagnostic biomarkers and 204 prognostic biomarkers. Among these biomarkers, 1,335 biomarkers came from serum samples, and 467 biomarkers came from plasma samples. In addition, metabolites from breath samples were used to diagnose lung cancer in seven studies [46, 48–53]. These studies identified 162 diagnostic markers with an AUC greater than or equal to 0.80. We also enriched the metabolic pathways of diagnostic metabolites in respiratory system cancers (Supplementary Figure 3). The main relevant pathways were aminoacyl-tRNA biosynthesis, arginine biosynthesis, glycine serine and threonine metabolism, and glyoxylate and dicarboxylate metabolism.

### Urinary system tumors

Urinary system cancer includes bladder cancer, kidney cancer, and ureteral cancer. Bladder cancer is known for its high recurrence rate worldwide [1, 40]. In this review, we collected 77 publications on urinary system tumors, encompassing 3 cancer types and 17 cancer subtypes. The sample size varied from 12 to 2,610 [54, 55]. The publications included 1,779 diagnostic biomarkers, 135 progression biomarkers, and 44 prognostic biomarkers. Urine samples were the primary diagnostic tool for urinary system cancers, with 51 articles including 1,483 biomarkers from urine used to detect these cancers. Additionally, there were 382 biomarkers from serum samples and 93 biomarkers from plasma samples. We identified 128 diagnostic markers with an AUC of 0.80 or higher. The enrichment analysis of metabolic pathways of diagnostic metabolites in urinary system cancer is presented in Supplementary Figure 4, showing that these metabolites were enriched in aminoacyl-tRNA biosynthesis, steroid hormone biosynthesis, glycine serine and threonine metabolism, and arginine biosynthesis.

### Nervous system tumors

Nervous system cancer, including brain cancer and other types of nervous system cancer, is one of the deadliest cancers, with 18,600 people in the United States dying from the disease in 2021 [40, 56]. Six cancer subtypes were reviewed in 16 articles, with sample sizes ranging from 17 to 220 [57, 58]. The studies included 223 diagnostic biomarkers, 31 progression biomarkers, and 13 prognostic biomarkers. Biomarkers were derived from serum samples (92 biomarkers), plasma samples (118 biomarkers), and cerebrospinal fluid samples (38 biomarkers). Of these studies, 24 diagnostic markers had an AUC of 0.80 or higher. A metabolic pathway enrichment analysis of diagnostic metabolites in nervous system cancers was conducted, and the four most relevant pathways were aminoacyl-tRNA biosynthesis, arginine biosynthesis, alanine aspartate and glutamate metabolism, and glyoxylate and dicarboxylate metabolism (Supplementary Figure 5).

### Other tumors

In addition to the aforementioned common cancers, abnormal metabolites have also been observed in other types of cancer. We collected 65 articles for the diagnosis of other cancers, including six cancer types (thyroid cancer, myeloma, leukemia, lymphoma, skin cancer, and osteosarcoma) and 24 cancer subtypes. The sample sizes ranged from 10 to 846 [59, 60]. Those studies included 1,393 diagnostic biomarkers, 27 progression biomarkers, and 97 prognostic biomarkers. Biomarkers were mainly derived from three types of samples: 725 biomarkers in serum samples, 586 biomarkers in plasma samples, and 105 biomarkers in urine samples. Those publications contained a total of 66 diagnostic markers with an AUC of 0.80 or higher.

## DISCUSSION

Cancer is one of the major threats to human health because of its high morbidity and mortality rates [61]. Highly specific and sensitive diagnostic or prognostic biomarkers can improve the efficiency of treatment and prolong the survival of patients [2]. Metabolomics has many exciting opportunities to promote the treatment of cancer [62]. For example, metabolomics combined with other “omics” can uncover valuable drug targets [63–65]. Metabolomics also has the potential to influence cancer screening and diagnosis. Since many studies have identified biomarkers in body fluids with high diagnostic value for human cancers [66, 67]. Zhou et al. reported that 4-Dodecylbenzenesulfonic acid, PC (30:1) and PC (44:5) were downregulated in the serum of colorectal adenoma patients compared to healthy subjects, with an AUC of 1.00 [68]. Plasma levels of beta-sitosterol were upregulated in pancreatic cancer patients compared to healthy individuals with an AUC value of 0.99 [69]. Plasma of hexadecasphinganine with an AUC value of 0.99 in the diagnosis of gastric cancer [70]. Serum levels of hypoxanthine were upregulated in patients with lung adenocarcinoma compared to normal controls with an AUC value of 0.99 [71]. Jové et al. found that hexanoic acid in the plasma had an AUC value of 1.00 for breast cancer diagnosis [72]. Metabolite pathway enrichment analysis is a good method to discover potential pathogenesis of different systemic cancers. Glycine serine and threonine metabolism and arginine biosynthesis were enriched in each system of cancer. These two metabolic pathways may provide inspiration for future cancer research.

Although many liquid biopsy biomarkers with high diagnostic value in human cancers have been reported, there are still difficulties and challenges in the clinical application of these metabolites. First, there is a lack of multi-center, large-scale studies to validate the clinical feasibility and reproducibility of metabolic markers [73]. Second, in order to incorporate biomarker assays into the clinical workflow, supporting assay resources, staff logistics, and technical education are needed, which can be costly in the clinic [73, 74]. Third, there are huge fluctuations in the concentration of metabolites *in vivo*, as well as a fragmented distribution of specialized small molecules in the body [75]. In addition, metabolomics is diverse and chemically complex, and varies in different tumor lesions. For example, L-alanine is significantly downregulated in pancreatic cancer but significantly upregulated in colorectal cancer, which adds great difficulty for tumor screening [7, 47, 76]. This article has some innovations that need to be clarified. First, previous studies mainly focused on biopsy markers of a single tumor and lacked a summary of diagnostic data on multiple tumor biopsies. This article summarizes diagnostic markers covering 24 tumor types. Second, our database contains more detailed information, such as AUC, accuracy, specificity, HMDB ID, metabolic pathway, sample source and so on. In addition, pathway analysis demonstrated that glycine, serine and threonine metabolism and arginine biosynthesis metabolic pathways were enriched in multiple cancer systems, suggesting that these two metabolic pathways play an important role in cancer diagnosis and treatment.

## CONCLUSION

With the development of standardized protocols, the measurement of metabolomics has become cheaper and more convenient. Metabolomics plays an increasingly important role in cancers, alongside other diagnostic and prognostic tests in the clinic. To provide an important resource for users to query, browse, and download information on cancer-related metabolites, we have established a user-friendly website.

## Supplementary Materials

Supplementary Figures

## References

[1] Sung H, Ferlay J, Siegel RL, Laversanne M, Soerjomataram I, Jemal A, Bray F. Global Cancer Statistics 2020: GLOBOCAN Estimates of Incidence and Mortality Worldwide for 36 Cancers in 185 Countries. CA Cancer J Clin. 2021; 71:209–49. 10.3322/caac.21660 33538338

[2] Crosby D, Bhatia S, Brindle KM, Coussens LM, Dive C, Emberton M, Esener S, Fitzgerald RC, Gambhir SS, Kuhn P, Rebbeck TR, Balasubramanian S. Early detection of cancer. Science. 2022; 375:eaay9040. 10.1126/science.aay9040 35298272

[3] Wender RC, Brawley OW, Fedewa SA, Gansler T, Smith RA. A blueprint for cancer screening and early detection: Advancing screening's contribution to cancer control. CA Cancer J Clin. 2019; 69:50–79. 10.3322/caac.21550 30452086

[4] Hackshaw A, Clarke CA, Hartman AR. New genomic technologies for multi-cancer early detection: Rethinking the scope of cancer screening. Cancer Cell. 2022; 40:109–13. 10.1016/j.ccell.2022.01.012 35120599

[5] Schiffman JD, Fisher PG, Gibbs P. Early detection of cancer: past, present, and future. Am Soc Clin Oncol Educ Book. 2015; 57–65. 10.14694/EdBook_AM.2015.35.57 25993143

[6] Newgard CB. Metabolomics and Metabolic Diseases: Where Do We Stand? Cell Metab. 2017; 25:43–56. 10.1016/j.cmet.2016.09.018 28094011 PMC5245686

[7] Schmidt DR, Patel R, Kirsch DG, Lewis CA, Vander Heiden MG, Locasale JW. Metabolomics in cancer research and emerging applications in clinical oncology. CA Cancer J Clin. 2021; 71:333–58. 10.3322/caac.21670 33982817 PMC8298088

[8] Rinschen MM, Ivanisevic J, Giera M, Siuzdak G. Identification of bioactive metabolites using activity metabolomics. Nat Rev Mol Cell Biol. 2019; 20:353–67. 10.1038/s41580-019-0108-4 30814649 PMC6613555

[9] Zamboni N, Saghatelian A, Patti GJ. Defining the metabolome: size, flux, and regulation. Mol Cell. 2015; 58:699–706. 10.1016/j.molcel.2015.04.021 26000853 PMC4831058

[10] Mader S, Pantel K. Liquid Biopsy: Current Status and Future Perspectives. Oncol Res Treat. 2017; 40:404–8. 10.1159/000478018 28693023

[11] Metallo CM, Vander Heiden MG. Understanding metabolic regulation and its influence on cell physiology. Mol Cell. 2013; 49:388–98. 10.1016/j.molcel.2013.01.018 23395269 PMC3569837

[12] González-Riano C, Dudzik D, Garcia A, Gil-de-la-Fuente A, Gradillas A, Godzien J, López-Gonzálvez Á, Rey-Stolle F, Rojo D, Ruperez FJ, Saiz J, Barbas C. Recent Developments along the Analytical Process for Metabolomics Workflows. Anal Chem. 2020; 92:203–26. 10.1021/acs.analchem.9b04553 31625723

[13] Rizzo C, Camarda F, Donzella D, La Barbera L, Guggino G. Metabolomics: An Emerging Approach to Understand Pathogenesis and to Assess Diagnosis and Response to Treatment in Spondyloarthritis. Cells. 2022; 11:549. 10.3390/cells11030549 35159358 PMC8834108

[14] Vander Heiden MG, DeBerardinis RJ. Understanding the Intersections between Metabolism and Cancer Biology. Cell. 2017; 168:657–69. 10.1016/j.cell.2016.12.039 28187287 PMC5329766

[15] Teicher BA, Linehan WM, Helman LJ. Targeting cancer metabolism. Clin Cancer Res. 2012; 18:5537–45. 10.1158/1078-0432.CCR-12-2587 23071355 PMC3475613

[16] Pavlova NN, Thompson CB. The Emerging Hallmarks of Cancer Metabolism. Cell Metab. 2016; 23:27–47. 10.1016/j.cmet.2015.12.006 26771115 PMC4715268

[17] Faubert B, Solmonson A, DeBerardinis RJ. Metabolic reprogramming and cancer progression. Science. 2020; 368:eaaw5473. 10.1126/science.aaw5473 32273439 PMC7227780

[18] Sreekumar A, Poisson LM, Rajendiran TM, Khan AP, Cao Q, Yu J, Laxman B, Mehra R, Lonigro RJ, Li Y, Nyati MK, Ahsan A, Kalyana-Sundaram S, et al. Metabolomic profiles delineate potential role for sarcosine in prostate cancer progression. Nature. 2009; 457:910–4. 10.1038/nature07762 19212411 PMC2724746

[19] Soga T, Sugimoto M, Honma M, Mori M, Igarashi K, Kashikura K, Ikeda S, Hirayama A, Yamamoto T, Yoshida H, Otsuka M, Tsuji S, Yatomi Y, et al. Serum metabolomics reveals γ-glutamyl dipeptides as biomarkers for discrimination among different forms of liver disease. J Hepatol. 2011; 55:896–905. 10.1016/j.jhep.2011.01.031 21334394

[20] Li J, Li J, Wang H, Qi LW, Zhu Y, Lai M. Tyrosine and Glutamine-Leucine Are Metabolic Markers of Early-Stage Colorectal Cancers. Gastroenterology. 2019; 157:257–9.e5. 10.1053/j.gastro.2019.03.020 30885779

[21] Wikoff WR, Hanash S, DeFelice B, Miyamoto S, Barnett M, Zhao Y, Goodman G, Feng Z, Gandara D, Fiehn O, Taguchi A. Diacetylspermine Is a Novel Prediagnostic Serum Biomarker for Non-Small-Cell Lung Cancer and Has Additive Performance With Pro-Surfactant Protein B. J Clin Oncol. 2015; 33:3880–6. 10.1200/JCO.2015.61.7779 26282655 PMC4652011

[22] Patel D, Thompson MD, Manna SK, Krausz KW, Zhang L, Nilubol N, Gonzalez FJ, Kebebew E. Unique and Novel Urinary Metabolomic Features in Malignant versus Benign Adrenal Neoplasms. Clin Cancer Res. 2017; 23:5302–10. 10.1158/1078-0432.CCR-16-3156 28450405 PMC5581680

[23] Wishart DS, Guo A, Oler E, Wang F, Anjum A, Peters H, Dizon R, Sayeeda Z, Tian S, Lee BL, Berjanskii M, Mah R, Yamamoto M, et al. HMDB 5.0: the Human Metabolome Database for 2022. Nucleic Acids Res. 2022; 50:D622–31. 10.1093/nar/gkab1062 34986597 PMC8728138

[24] Goodman RP, Markhard AL, Shah H, Sharma R, Skinner OS, Clish CB, Deik A, Patgiri A, Hsu YH, Masia R, Noh HL, Suk S, Goldberger O, et al. Hepatic NADH reductive stress underlies common variation in metabolic traits. Nature. 2020; 583:122–6. 10.1038/s41586-020-2337-2 32461692 PMC7536642

[25] Zhu G, Wang S, Huang Z, Zhang S, Liao Q, Zhang C, Lin T, Qin M, Peng M, Yang C, Cao X, Han X, Wang X, et al. Rewiring of the Fruit Metabolome in Tomato Breeding. Cell. 2018; 172:249–61.e12. 10.1016/j.cell.2017.12.019 29328914

[26] Medina CB, Mehrotra P, Arandjelovic S, Perry JSA, Guo Y, Morioka S, Barron B, Walk SF, Ghesquière B, Krupnick AS, Lorenz U, Ravichandran KS. Metabolites released from apoptotic cells act as tissue messengers. Nature. 2020; 580:130–5. 10.1038/s41586-020-2121-3 32238926 PMC7217709

[27] Zhang Z, Du C, de Barsy F, Liem M, Liakopoulos A, van Wezel GP, Choi YH, Claessen D, Rozen DE. Antibiotic production in *Streptomyces* is organized by a division of labor through terminal genomic differentiation. Sci Adv. 2020; 6:eaay5781. 10.1126/sciadv.aay5781 31998842 PMC6962034

[28] Wishart DS. Metabolomics for Investigating Physiological and Pathophysiological Processes. Physiol Rev. 2019; 99:1819–75. 10.1152/physrev.00035.2018 31434538

[29] Ducker GS, Ghergurovich JM, Mainolfi N, Suri V, Jeong SK, Hsin-Jung Li S, Friedman A, Manfredi MG, Gitai Z, Kim H, Rabinowitz JD. Human SHMT inhibitors reveal defective glycine import as a targetable metabolic vulnerability of diffuse large B-cell lymphoma. Proc Natl Acad Sci U S A. 2017; 114:11404–9. 10.1073/pnas.1706617114 29073064 PMC5664509

[30] Mills EL, Pierce KA, Jedrychowski MP, Garrity R, Winther S, Vidoni S, Yoneshiro T, Spinelli JB, Lu GZ, Kazak L, Banks AS, Haigis MC, Kajimura S, et al. Accumulation of succinate controls activation of adipose tissue thermogenesis. Nature. 2018; 560:102–6. 10.1038/s41586-018-0353-2 30022159 PMC7045287

[31] Griffin JL, Shockcor JP. Metabolic profiles of cancer cells. Nat Rev Cancer. 2004; 4:551–61. 10.1038/nrc1390 15229480

[32] Cairns RA, Harris IS, Mak TW. Regulation of cancer cell metabolism. Nat Rev Cancer. 2011; 11:85–95. 10.1038/nrc2981 21258394

[33] Hu J, Locasale JW, Bielas JH, O'Sullivan J, Sheahan K, Cantley LC, Vander Heiden MG, Vitkup D. Heterogeneity of tumor-induced gene expression changes in the human metabolic network. Nat Biotechnol. 2013; 31:522–9. 10.1038/nbt.2530 23604282 PMC3681899

[34] Bamji-Stocke S, van Berkel V, Miller DM, Frieboes HB. A review of metabolism-associated biomarkers in lung cancer diagnosis and treatment. Metabolomics. 2018; 14:81. 10.1007/s11306-018-1376-2 29983671 PMC6033515

[35] Günther UL. Metabolomics Biomarkers for Breast Cancer. Pathobiology. 2015; 82:153–65. 10.1159/000430844 26330356

[36] Kdadra M, Höckner S, Leung H, Kremer W, Schiffer E. Metabolomics Biomarkers of Prostate Cancer: A Systematic Review. Diagnostics (Basel). 2019; 9:21. 10.3390/diagnostics9010021 30791464 PMC6468767

[37] Erben V, Bhardwaj M, Schrotz-King P, Brenner H. Metabolomics Biomarkers for Detection of Colorectal Neoplasms: A Systematic Review. Cancers (Basel). 2018; 10:246. 10.3390/cancers10080246 30060469 PMC6116151

[38] Wishart DS. Emerging applications of metabolomics in drug discovery and precision medicine. Nat Rev Drug Discov. 2016; 15:473–84. 10.1038/nrd.2016.32 26965202

[39] Bi J, Chowdhry S, Wu S, Zhang W, Masui K, Mischel PS. Altered cellular metabolism in gliomas - an emerging landscape of actionable co-dependency targets. Nat Rev Cancer. 2020; 20:57–70. 10.1038/s41568-019-0226-5 31806884

[40] Siegel RL, Miller KD, Fuchs HE, Jemal A. Cancer statistics, 2022. CA Cancer J Clin. 2022; 72:7–33. 10.3322/caac.21708 35020204

[41] Winter H, Kaisaki PJ, Harvey J, Giacopuzzi E, Ferla MP, Pentony MM, Knight SJL, Sharma RA, Taylor JC, McCullagh JSO. Identification of Circulating Genomic and Metabolic Biomarkers in Intrahepatic Cholangiocarcinoma. Cancers (Basel). 2019; 11:1895. 10.3390/cancers11121895 31795195 PMC6966597

[42] Jia W, Xie G. Probiotics, bile acids and gastrointestinal carcinogenesis. Nat Rev Gastroenterol Hepatol. 2018; 15:205. 10.1038/nrgastro.2018.24 29512647 PMC5898972

[43] Jia W, Xie G, Jia W. Bile acid-microbiota crosstalk in gastrointestinal inflammation and carcinogenesis. Nat Rev Gastroenterol Hepatol. 2018; 15:111–28. 10.1038/nrgastro.2017.119 29018272 PMC5899973

[44] Audet-Delage Y, Villeneuve L, Grégoire J, Plante M, Guillemette C. Identification of Metabolomic Biomarkers for Endometrial Cancer and Its Recurrence after Surgery in Postmenopausal Women. Front Endocrinol (Lausanne). 2018; 9:87. 10.3389/fendo.2018.00087 29593653 PMC5857535

[45] Schmidt JA, Fensom GK, Rinaldi S, Scalbert A, Appleby PN, Achaintre D, Gicquiau A, Gunter MJ, Ferrari P, Kaaks R, Kühn T, Boeing H, Trichopoulou A, et al. Patterns in metabolite profile are associated with risk of more aggressive prostate cancer: A prospective study of 3,057 matched case-control sets from EPIC. Int J Cancer. 2020; 146:720–30. 10.1002/ijc.32314 30951192 PMC6916595

[46] Xu H, Wei Y, Zhu L, Huang J, Li Y, Liu F, Wang S, Liu S. Bifunctional magnetic nanoparticles for analysis of aldehyde metabolites in exhaled breath of lung cancer patients. J Chromatogr A. 2014; 1324:29–35. 10.1016/j.chroma.2013.11.041 24315678

[47] Miyagi Y, Higashiyama M, Gochi A, Akaike M, Ishikawa T, Miura T, Saruki N, Bando E, Kimura H, Imamura F, Moriyama M, Ikeda I, Chiba A, et al. Plasma free amino acid profiling of five types of cancer patients and its application for early detection. PLoS One. 2011; 6:e24143. 10.1371/journal.pone.0024143 21915291 PMC3168486

[48] Koureas M, Kirgou P, Amoutzias G, Hadjichristodoulou C, Gourgoulianis K, Tsakalof A. Target Analysis of Volatile Organic Compounds in Exhaled Breath for Lung Cancer Discrimination from Other Pulmonary Diseases and Healthy Persons. Metabolites. 2020; 10:317. 10.3390/metabo10080317 32756521 PMC7464039

[49] Peralbo-Molina A, Calderón-Santiago M, Priego-Capote F, Jurado-Gámez B, Luque de Castro MD. Identification of metabolomics panels for potential lung cancer screening by analysis of exhaled breath condensate. J Breath Res. 2016; 10:026002. 10.1088/1752-7155/10/2/026002 27007686

[50] Peralbo-Molina A, Calderón-Santiago M, Priego-Capote F, Jurado-Gámez B, Luque de Castro MD. Metabolomics analysis of exhaled breath condensate for discrimination between lung cancer patients and risk factor individuals. J Breath Res. 2016; 10:016011. 10.1088/1752-7155/10/1/016011 26866403

[51] Huang J, Deng H, Song D, Xu H. Electrospun polystyrene/graphene nanofiber film as a novel adsorbent of thin film microextraction for extraction of aldehydes in human exhaled breath condensates. Anal Chim Acta. 2015; 878:102–8. 10.1016/j.aca.2015.03.053 26002331

[52] Wang C, Dong R, Wang X, Lian A, Chi C, Ke C, Guo L, Liu S, Zhao W, Xu G, Li E. Exhaled volatile organic compounds as lung cancer biomarkers during one-lung ventilation. Sci Rep. 2014; 4:7312. 10.1038/srep07312 25482491 PMC4258651

[53] Filipiak W, Filipiak A, Sponring A, Schmid T, Zelger B, Ager C, Klodzinska E, Denz H, Pizzini A, Lucciarini P, Jamnig H, Troppmair J, Amann A. Comparative analyses of volatile organic compounds (VOCs) from patients, tumors and transformed cell lines for the validation of lung cancer-derived breath markers. J Breath Res. 2014; 8:027111. 10.1088/1752-7155/8/2/027111 24862102

[54] Ganti S, Taylor SL, Kim K, Hoppel CL, Guo L, Yang J, Evans C, Weiss RH. Urinary acylcarnitines are altered in human kidney cancer. Int J Cancer. 2012; 130:2791–800. 10.1002/ijc.26274 21732340 PMC3258465

[55] Guida F, Tan VY, Corbin LJ, Smith-Byrne K, Alcala K, Langenberg C, Stewart ID, Butterworth AS, Surendran P, Achaintre D, Adamski J, Amiano P, Bergmann MM, et al. The blood metabolome of incident kidney cancer: A case-control study nested within the MetKid consortium. PLoS Med. 2021; 18:e1003786. 10.1371/journal.pmed.1003786 34543281 PMC8496779

[56] Miller KD, Ostrom QT, Kruchko C, Patil N, Tihan T, Cioffi G, Fuchs HE, Waite KA, Jemal A, Siegel RL, Barnholtz-Sloan JS. Brain and other central nervous system tumor statistics, 2021. CA Cancer J Clin. 2021; 71:381–406. 10.3322/caac.21693 34427324

[57] Locasale JW, Melman T, Song S, Yang X, Swanson KD, Cantley LC, Wong ET, Asara JM. Metabolomics of human cerebrospinal fluid identifies signatures of malignant glioma. Mol Cell Proteomics. 2012; 11:M111.014688. 10.1074/mcp.M111.014688 22240505 PMC3433896

[58] Björkblom B, Wibom C, Jonsson P, Mörén L, Andersson U, Johannesen TB, Langseth H, Antti H, Melin B. Metabolomic screening of pre-diagnostic serum samples identifies association between α- and γ-tocopherols and glioblastoma risk. Oncotarget. 2016; 7:37043–53. 10.18632/oncotarget.9242 27175595 PMC5095057

[59] Rad Pour S, Morikawa H, Kiani NA, Gomez-Cabrero D, Hayes A, Zheng X, Pernemalm M, Lehtiö J, Mole DJ, Hansson J, Eriksson H, Tegnér J. Immunometabolic Network Interactions of the Kynurenine Pathway in Cutaneous Malignant Melanoma. Front Oncol. 2020; 10:51. 10.3389/fonc.2020.00051 32117720 PMC7017805

[60] Chen WL, Wang JH, Zhao AH, Xu X, Wang YH, Chen TL, Li JM, Mi JQ, Zhu YM, Liu YF, Wang YY, Jin J, Huang H, et al. A distinct glucose metabolism signature of acute myeloid leukemia with prognostic value. Blood. 2014; 124:1645–54. 10.1182/blood-2014-02-554204 25006128 PMC5726328

[61] Bradner JE, Hnisz D, Young RA. Transcriptional Addiction in Cancer. Cell. 2017; 168:629–43. 10.1016/j.cell.2016.12.013 28187285 PMC5308559

[62] DeBerardinis RJ, Keshari KR. Metabolic analysis as a driver for discovery, diagnosis, and therapy. Cell. 2022; 185:2678–89. 10.1016/j.cell.2022.06.029 35839759 PMC9469798

[63] Fathi AT, Sadrzadeh H, Borger DR, Ballen KK, Amrein PC, Attar EC, Foster J, Burke M, Lopez HU, Matulis CR, Edmonds KM, Iafrate AJ, Straley KS, et al. Prospective serial evaluation of 2-hydroxyglutarate, during treatment of newly diagnosed acute myeloid leukemia, to assess disease activity and therapeutic response. Blood. 2012; 120:4649–52. 10.1182/blood-2012-06-438267 23074281

[64] Dang L, White DW, Gross S, Bennett BD, Bittinger MA, Driggers EM, Fantin VR, Jang HG, Jin S, Keenan MC, Marks KM, Prins RM, Ward PS, et al. Cancer-associated IDH1 mutations produce 2-hydroxyglutarate. Nature. 2009; 462:739–44. 10.1038/nature08617 19935646 PMC2818760

[65] Ward PS, Patel J, Wise DR, Abdel-Wahab O, Bennett BD, Coller HA, Cross JR, Fantin VR, Hedvat CV, Perl AE, Rabinowitz JD, Carroll M, Su SM, et al. The common feature of leukemia-associated IDH1 and IDH2 mutations is a neomorphic enzyme activity converting alpha-ketoglutarate to 2-hydroxyglutarate. Cancer Cell. 2010; 17:225–34. 10.1016/j.ccr.2010.01.020 20171147 PMC2849316

[66] Mayers JR, Wu C, Clish CB, Kraft P, Torrence ME, Fiske BP, Yuan C, Bao Y, Townsend MK, Tworoger SS, Davidson SM, Papagiannakopoulos T, Yang A, et al. Elevation of circulating branched-chain amino acids is an early event in human pancreatic adenocarcinoma development. Nat Med. 2014; 20:1193–8. 10.1038/nm.3686 25261994 PMC4191991

[67] Liu X, Romero IL, Litchfield LM, Lengyel E, Locasale JW. Metformin Targets Central Carbon Metabolism and Reveals Mitochondrial Requirements in Human Cancers. Cell Metab. 2016; 24:728–39. 10.1016/j.cmet.2016.09.005 27746051 PMC5889952

[68] Zhou H, Nong Y, Zhu Y, Liang Y, Zhang J, Chen H, Zhu P, Zhang Q. Serum untargeted lipidomics by UHPLC-ESI-HRMS aids the biomarker discovery of colorectal adenoma. BMC Cancer. 2022; 22:314. 10.1186/s12885-022-09427-1 35331175 PMC8943952

[69] Luo X, Liu J, Wang H, Lu H. Metabolomics identified new biomarkers for the precise diagnosis of pancreatic cancer and associated tissue metastasis. Pharmacol Res. 2020; 156:104805. 10.1016/j.phrs.2020.104805 32278036

[70] Yu L, Lai Q, Feng Q, Li Y, Feng J, Xu B. Serum Metabolic Profiling Analysis of Chronic Gastritis and Gastric Cancer by Untargeted Metabolomics. Front Oncol. 2021; 11:636917. 10.3389/fonc.2021.636917 33777793 PMC7991914

[71] Yu M, Sun R, Zhao Y, Shao F, Zhu W, Aa J. Detection and verification of coexisting diagnostic markers in plasma and serum of patients with non-small-cell lung cancer. Future Oncol. 2021; 17:4355–69. 10.2217/fon-2021-0025 34674559

[72] Jové M, Collado R, Quiles JL, Ramírez-Tortosa MC, Sol J, Ruiz-Sanjuan M, Fernandez M, de la Torre Cabrera C, Ramírez-Tortosa C, Granados-Principal S, Sánchez-Rovira P, Pamplona R. A plasma metabolomic signature discloses human breast cancer. Oncotarget. 2017; 8:19522–33. 10.18632/oncotarget.14521 28076849 PMC5386702

[73] Ignatiadis M, Sledge GW, Jeffrey SS. Liquid biopsy enters the clinic - implementation issues and future challenges. Nat Rev Clin Oncol. 2021; 18:297–312. 10.1038/s41571-020-00457-x 33473219

[74] Dinges SS, Hohm A, Vandergrift LA, Nowak J, Habbel P, Kaltashov IA, Cheng LL. Cancer metabolomic markers in urine: evidence, techniques and recommendations. Nat Rev Urol. 2019; 16:339–62. 10.1038/s41585-019-0185-3 31092915

[75] Böttcher T. From Molecules to Life: Quantifying the Complexity of Chemical and Biological Systems in the Universe. J Mol Evol. 2018; 86:1–10. 10.1007/s00239-017-9824-6 29260254 PMC5794832

[76] Michálková L, Horník Š, Sýkora J, Habartová L, Setnička V, Bunganič B. Early Detection of Pancreatic Cancer in Type 2 Diabetes Mellitus Patients Based on ^1^H NMR Metabolomics. J Proteome Res. 2021; 20:1744–53. 10.1021/acs.jproteome.0c00990 33617266

