# Supplementary material for: HCMMD: systematic evaluation of metabolites in body fluids as liquid biopsy biomarker for human cancers
Source: Aging (Albany NY). 2024 Apr 26;16(8):7487–504. doi: 10.18632/aging.205779 (PMC11087094; doi:10.18632/aging.205779)
Supplement: Supplementary Figures [file aging-16-205779-s001.pdf]

SUPPLEMENTARY FIGURES

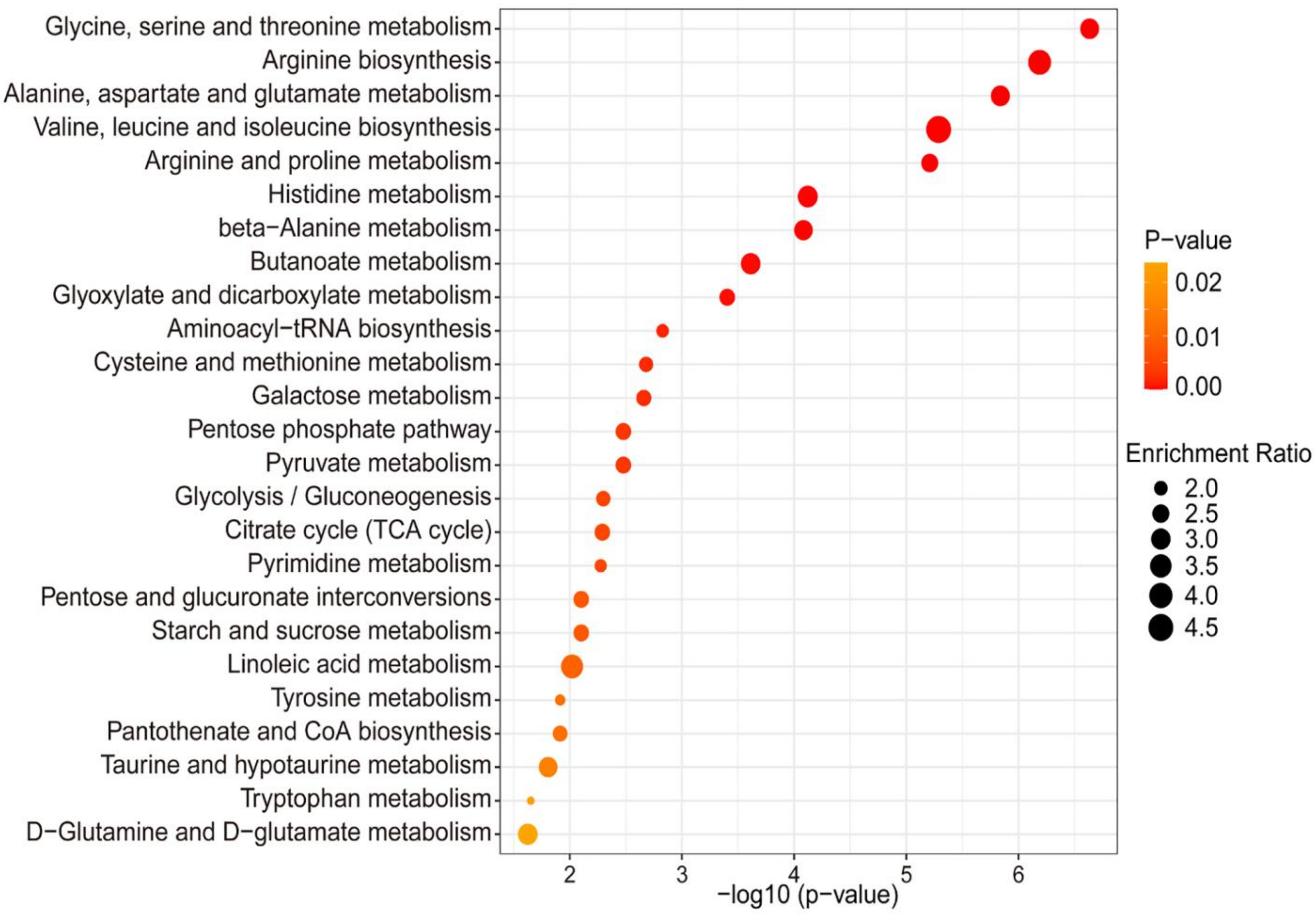

Supplementary Figure 1. Top 25 metabolic pathways for diagnostic metabolite enrichment analysis in digestive system tumors.

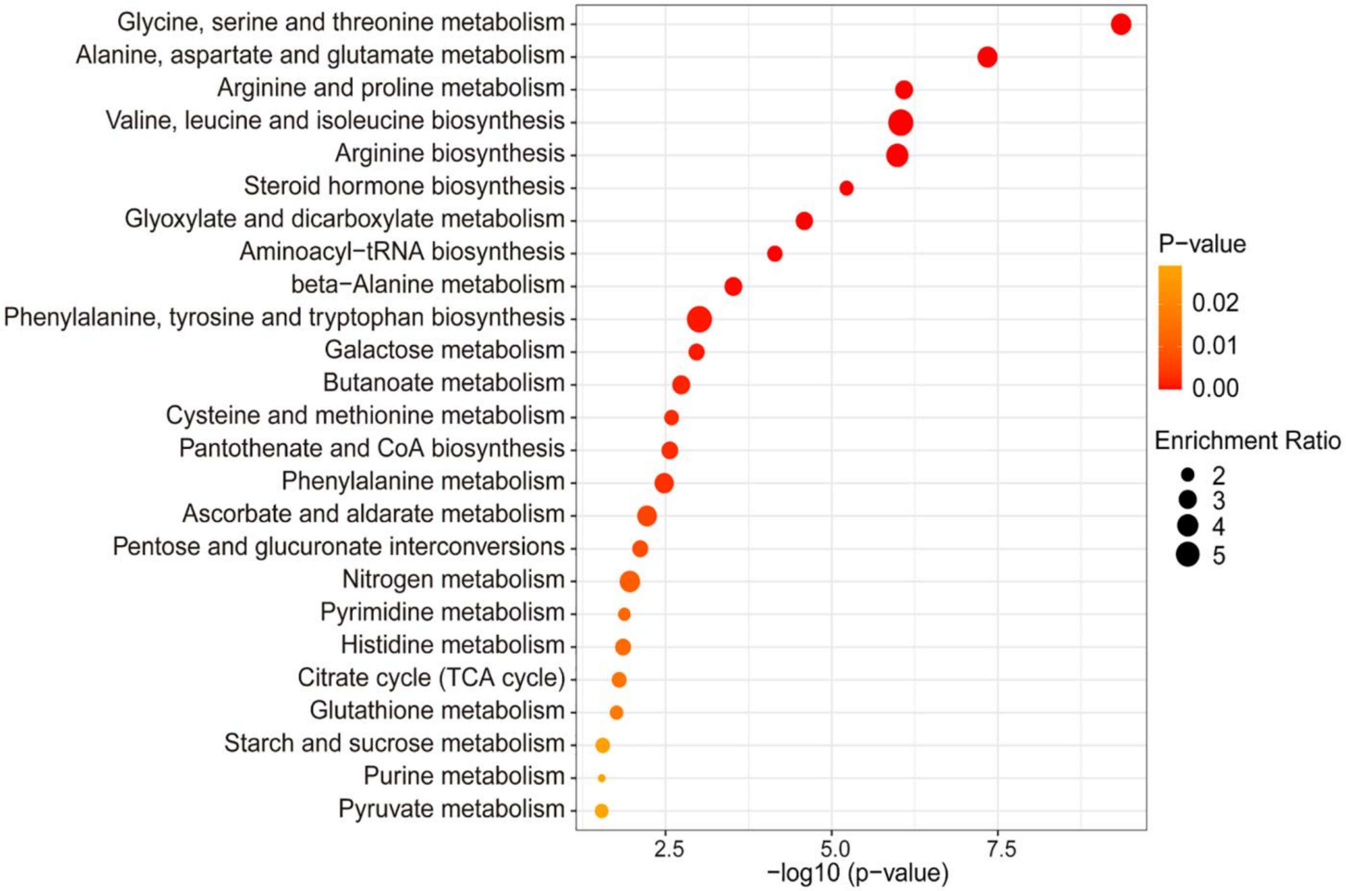

Supplementary Figure 2. Top 25 metabolic pathways for diagnostic metabolite enrichment analysis in reproductive system tumors.

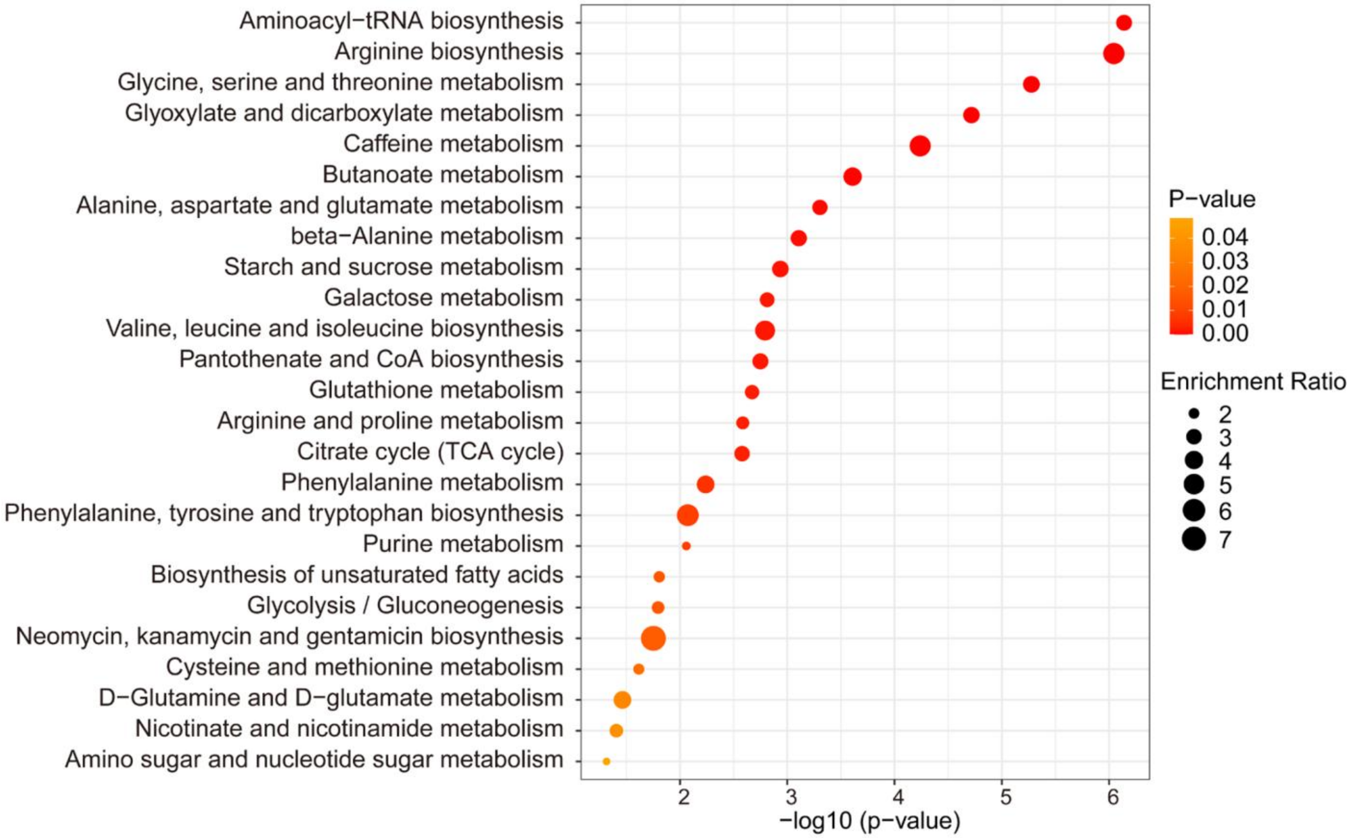

Supplementary Figure 3. Top 25 metabolic pathways for diagnostic metabolite enrichment analysis in respiratory system tumors.

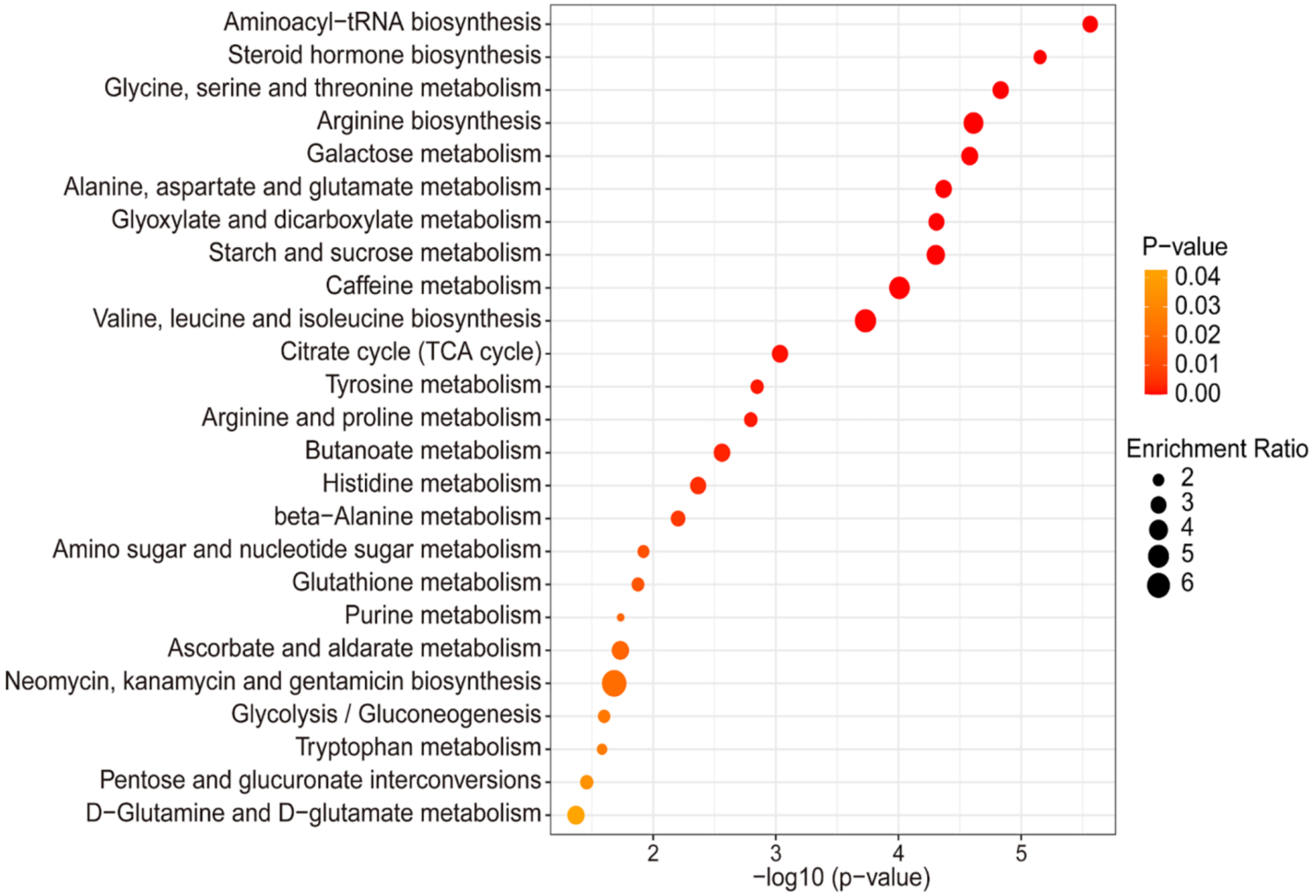

Supplementary Figure 4. Top 25 metabolic pathways for diagnostic metabolite enrichment analysis in urinary system tumors

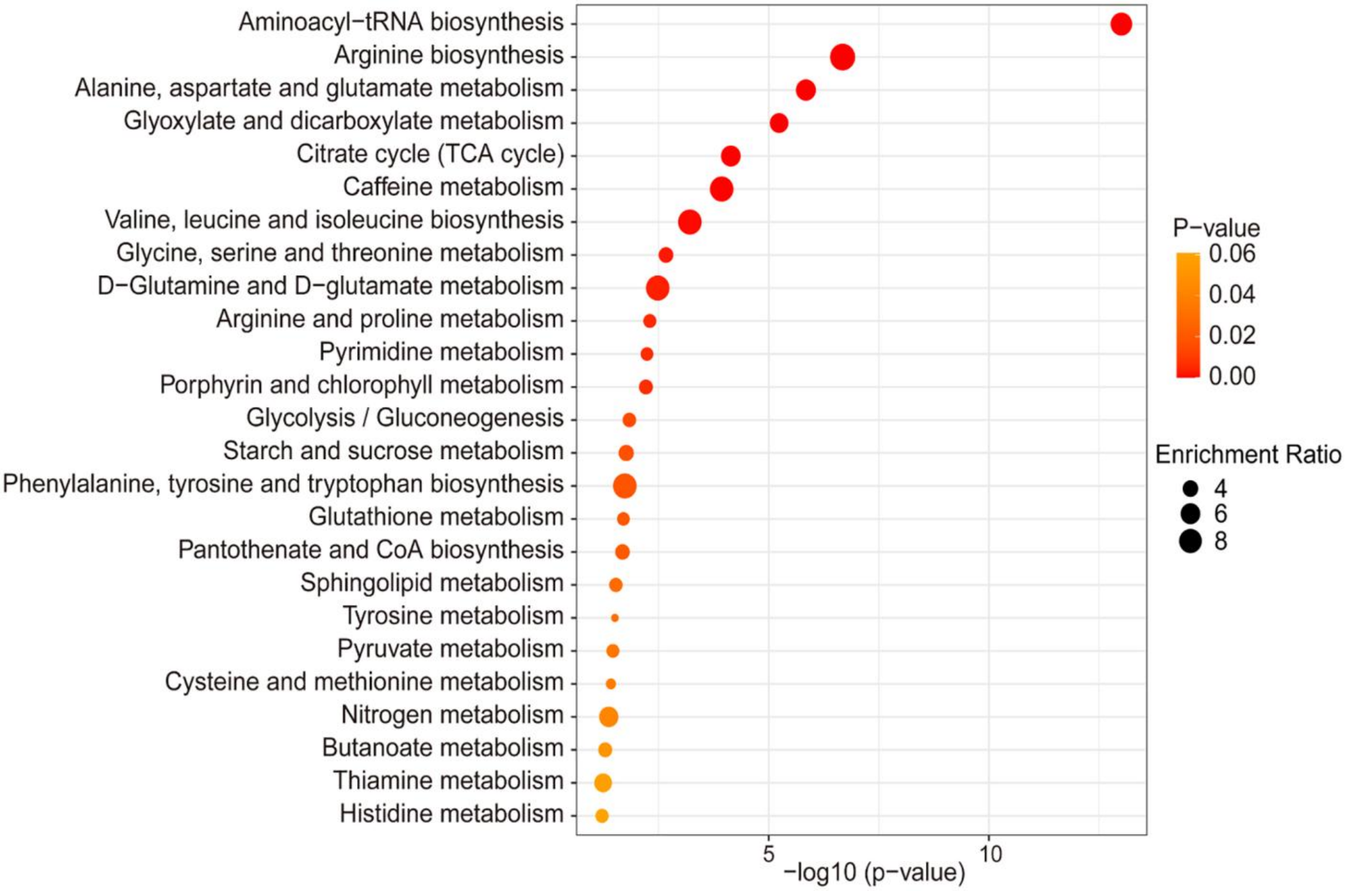

Supplementary Figure 5. Top 25 metabolic pathways for diagnostic metabolite enrichment analysis in nervous system tumors.
